# Scenario Factory 2.0: Scenario-Based Testing of Automated Vehicles with CommonRoad

**DOI:** 10.1007/s42154-025-00360-0

**Published:** 2025-05-25

**Authors:** Florian Finkeldei, Christoph Thees, Jan-Niklas Weghorn, Matthias Althoff

**Affiliations:** TUM School of Computation, Information and Technology (CIT), Technical University of Munich, Boltzmannstr. 3, 85748 Garching, Germany

**Keywords:** Automated vehicles, Autonomous driving safety, Microscopic traffic simulation, Scenario-based testing, Scenario Factory 2.0, CommonRoad, OpenTrafficSim, SUMO

## Abstract

Scenario-based testing plays a pivotal role in the development and validation of automated vehicles. Its main challenge is to efficiently generate realistic and relevant test scenarios to identify and analyze shortcomings of automated driving systems. The Scenario Factory 2.0 unifies several scenario generation techniques from the open-source CommonRoad framework and introduces simulation modes for coupling with the traffic simulators OpenTrafficSim and SUMO. The simulation modes enable generating scenarios with a tunable similarity to existing ones. As existing approaches, the Scenario Factory 2.0 integrates scenario generation from formal specifications and falsification techniques. Scenario Factory 2.0 has a modular structure and the modules can be easily rearranged for creating required scenarios. We evaluate the effectiveness of the novel simulation modes for various traffic scenarios and demonstrate the scenario generation with Scenario Factory 2.0 in a use case. The open-source code is provided at https://commonroad.in.tum.de/tools/scenario-factory.

## Introduction

Automated driving systems aim to enhance traffic safety compared to human drivers. The UN [1] and EU [2] regulations rely on scenario-based testing to assess the safety of automated driving systems and include scenario specifications and acceptance conditions. While these specified scenarios cover important safety aspects, the development of safe autonomous vehicles requires additional testing, placing several requirements on the testing scenarios and their generation process.

It is desirable that required scenarios can be automatically generated and that they are realistic, i.e., likely to occur in reality (see Sec. II-C [3]). In addition, the scenario generation process should be controllable, meaning that resulting scenarios show a specified behavior (see Sec. VII [3]). With specification-compliant scenarios, one can systematically analyze the behavior of automated driving systems. To detect unknown shortcomings and explore the robustness of automated driving systems in diverse traffic situations, the deliberate generation of deviating scenarios is desirable (see Sec. VII-E [3]). Furthermore, a tunable criticality of traffic scenarios enables targeted testing in critical situations (see Sec. VII [3]). Additionally, the overall scenario generation process should be computationally efficient (see Sec. VIII-A [4]).

To address these requirements, numerous scenario generation techniques with individual performance characteristics emerged (see Sec. 2). Combining the various techniques promises to obtain more relevant test scenarios more efficiently and to leverage synergies: For example, scenarios synthesized from formal specifications can be precisely controlled but might not be sufficiently realistic. By subsequently simulating similar scenarios using high-fidelity human driver models, the behavior can be specifically adapted towards reality. However, being realized by specialized tools with deviating data formats, the various scenario generation techniques are difficult to combine with each other.

This article presents Scenario Factory 2.0, a framework that realizes the entire pipeline from automatic map generation to generating critical scenarios: It bundles and integrates existing scenario syntheses from the CommonRoad ecosystem and introduces simulation modes that make microscopic traffic simulation applicable in different stages of the scenario generation process. Scenario Factory 2.0 further supports researchers with utilities, such as automatic map creation from OpenStreetMap data, scenario labeling and criticality assessment, testing of motion planners, and visualization. Our main contributions are:

- Introduction of a data structure and processing pipeline to flexibly combine scenario generation techniques.
- Integrating the functionalities of several existing scenario generation techniques.
- Users can generate scenarios from existing ones with a tunable similarity through the novel simulation modes using SUMO and OpenTrafficSim.

The remainder of this article is structured as follows: Sec. 2 summarizes related work. Subsequently, we present Scenario Factory 2.0 in Sec. 3 and introduce the implemented simulation modes in Sec. 4. The effectiveness of the simulation modes using the OpenTrafficSim and SUMO couplings is demonstrated, and a use case of Scenario Factory 2.0 is shown in Sec. 5. The article concludes in Sec. 6.

## Related Work

We survey and classify tools and techniques to provide test cases for scenario-based testing. In particular, we provide an overview of microscopic traffic simulation, as this will be especially relevant to our novel simulation modes.

### Scenario Generation

The literature distinguishes scenarios generated from data, artificial intelligence, and knowledge (see Sec. II-C [4]). Data-driven approaches sample scenarios from a database of traffic recordings [3, Sec. III]. While recordings are obviously realistic, the diversity of extracted scenarios is limited to the recordings and can only be controlled by a selection process (see Sec. IV [4]). Moreover, collecting the database is time-consuming and costly, as it can only be partly automated and requires special hardware (see Ref. [5], and Sec. VI-A [3]).

A different method to utilize traffic datasets is to use generative artificial intelligence. Several works apply this approach to generate a wide range of traffic scenarios [6], including lane changes [7] and accidents [8]. While providing realistic and efficient scenario generation, the controllability of these approaches is typically limited. Methodically closely connected are approaches from the Waymo motion prediction challenge [9], where the trajectories of agents are predicted for 8 s based on a 1 s history and maps. While recent approaches achieve high motion prediction accuracy [10], they do not explicitly offer a high diversity and controllability.

Knowledge-based scenario generation relies on provided knowledge, such as traffic rules, physical models, and formal specifications [3, 4]. It is often efficient and computationally cheap (see Sec. VII [3]). Depending on the specific type of knowledge-based scenario synthesis, the resulting traffic scenarios can be diverse, and their properties are controllable (see Sec. VII [3]). Conversely, the achieved realism might be limited (see Sec. VII [3]). In microscopic traffic simulation [11], surveyed in Sec. 2.2, knowledge is provided through human driver models. One approach for deriving traffic scenarios from formal specifications is using numerical optimization, with the specifications being modeled as linear inequality constraints [12, 13]. Formal specifications are also used in falsification or adversarial techniques: These approaches adjust traffic scenarios to provoke failures of the vehicle under test (see Sec. IV [3]). While these approaches are efficient in detecting shortcomings of automated driving systems, they are often limited in realism and diversity (see Sec. VII [3]). Another way for modeling knowledge are ontologies. These are first converted into logical scenarios [14], which serve as a starting point for synthesizing concrete scenarios [15].

Combinations of existing approaches can overcome their respective, individual weaknesses (see Sec. VIII-C-1 [4]). Following this idea, a combination of knowledge-based and machine learning approaches is applied in Ref. [16], which focuses on the interactive simulation of traffic scenarios. Various works combine data-driven and knowledge-based approaches: The authors of Ref. [17] combine traffic simulation tools with recorded traffic data by fitting the parameter distributions of simulated vehicles accordingly. The SceGene tool applies evolutionary mutations to recombine scenario features of recorded scenarios to create relevant scenarios [18]. As this might lead to dynamically non-feasible trajectories, the scenarios are repaired using human driver models from microscopic traffic simulation.

Recently, tools emerged that specifically assist researchers and developers in the scenario creation process. Scenario-Net [19] utilizes TrafficGen [6] to create new traffic scenarios. It extends TrafficGen by adding support for multiple open-source datasets and rendering in the MetaDrive simulator [20]. LimSim [21] integrates flow-based, vehicle-based, and data-based simulation techniques to enhance simulation accuracy and stability. It also provides scenario analysis functionalities. The tool scenario.center [22] analyzes existing traffic data to create a searchable database. Given requirements of the user, the scenarios are provided as real-world recordings, interactive simulations, or logical scenarios.

### Microscopic Traffic Simulation

In microscopic traffic simulations, traffic participants are simulated individually (see p.15 [23]) on a road network using human driver models that respond to surrounding traffic (see p.18 [23]). To model the topology of road networks, graphs with edges representing the roads are used (see p. 273 [24]). Lanelet-based approaches extend the graph topology by considering the geometry of the lanes and including references to, e.g., preceding or adjacent lanelets [25, 26]. The road network information is complemented by additional information, such as traffic signs, traffic lights, and obstacles. The conversion between different road infrastructure representations is generally possible without or with minor loss of information [27]. Human driver models are typically divided into two levels: the strategical level predicts the route choice, whereas the tactical one models the motion [24, 28]. The tactical level is often split into longitudinal and lateral motion, e.g., vehicle following and lane changing models.

Vehicle following models typically provide the acceleration given the current traffic situation (see Ref. [29], and p. 18 [23]). One implementation of a vehicle following model is the intelligent driver model+ (IDM+). The acceleration function of the IDM+ multiplies the comfortable acceleration *a* with the minimum of velocity- and gap-correction terms that consider deviations from the desired speed $v_0$ and the minimum gap $s^*$ (see Eq.(3) [30]):

1 $$\begin{aligned} \dot{v} = a \cdot \min \left( 1-\left( \frac{v}{v_0}\right) ^4, 1-\left( \frac{s^*}{s}\right) ^2 \right) \end{aligned}$$

In free-flow conditions, the velocity term ensures that the current speed *v* converges to the desired speed $v_0$. The gap term avoids that the current gap to the preceding vehicle *s* falls below a desired minimum value $s^*$. This desired minimum gap $s^*$ additionally depends on the safety gap $s_0$ and safety time gap *T*, the velocity difference to the preceding vehicle $\Delta v$, and the comfortable deceleration *b* (see Eq.(3) [29]):

2 $$\begin{aligned} s^* = s_0 + v \cdot T + \frac{v \cdot \Delta v}{2 \sqrt{a \cdot b}} \end{aligned}$$

The IDM+ is an extension to the well known IDM with improved accuracy in situations with high traffic flows *Q* [30], which is defined as the inverse of the time headway (see Eq. (12) [29]) with vehicle length *l*:

3 $$\begin{aligned} Q = \frac{v}{s+l} \end{aligned}$$

An overview of currently available microscopic traffic simulators is provided in Ref. [31]. Two of them are integrated in Scenario Factory 2.0: SUMO is a well established toolbox for multimodal traffic simulations [24]. OpenTrafficSim uses state-of-the-art human driver models [30, 32], including cognitive and perception models.

## Scenario Factory 2.0

Scenario Factory 2.0 provides numerous features for scenario-based testing of automated driving systems. We first introduce the CommonRoad scenario format, available traffic datasets, and the CommonRoad scenario database (Sec. 3.1). Next, the scenario generation and adaptation techniques supported by Scenario Factory 2.0 are explained, which includes traffic simulation (Sec. 3.2), specification-compliant scenario generation (Sec. 3.3), and falsification (Sec. 3.4). Subsequently, the modular software architecture of Scenario Factory 2.0 is presented (Sec. 3.5).

### CommonRoad Scenarios

CommonRoad scenarios contain meta information, a road network, vehicle representations, and planning problems [25]. Meta information comprises, e.g., a unique benchmark ID, date, author, source, location, tags, and the time step size of a scenario. Lanelet networks represent road networks in CommonRoad [25]. A lanelet is defined by arrays of vertices of its left and right bounds, adjacency to other lanelets, line markings, allowed user types, and references to traffic signs and lights [25]. The CommonRoad framework features dataset converters for many publicly available traffic datasets. Moreover, it provides the MONA dataset with an extensive processing framework published under an open-source license [33]. Generated or recorded traffic scenarios can be stored in a scenario database and accessed using the scenario selection tool.^1^ The selection tool enables to filter scenarios based on, e.g., assigned tags, duration, and properties of obstacles and planning problems.

### Traffic Simulation

**Fig. 1:**
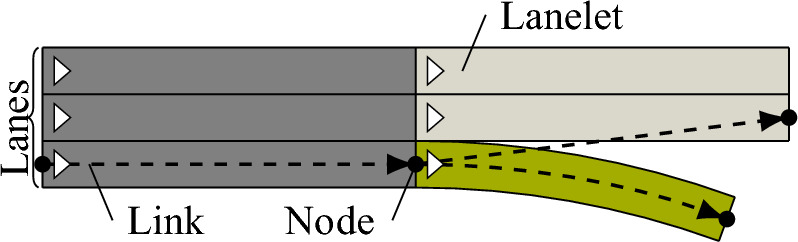
Conversion of a CommonRoad lanelet network to an OpenTrafficSim graph; same fill color: one link with possibly several lanes

With the SUMO and OpenTrafficSim coupling, one can generate scenarios using microscopic traffic simulations. Both tools use directed graphs to describe the road network. Subsequently, the map conversion for OpenTrafficSim is described. The conversion for SUMO is explained  in Ref. [34]. In OpenTrafficSim, so-called links represent roads between the nodes of a graph. To model multi-lane roads, lanes specify the lateral segmentation of links (see Fig. 1). Lanes provide a centerline and shape that are taken from their corresponding CommonRoad lanelets. When converting CommonRoad lanelet networks to OpenTrafficSim graphs, adjacent lanelets with the same driving direction are grouped as one link with several lanes (same color in Fig. 1). Details on the conversion of traffic signs and lights are provided in Appendix B.1. The simulation modes that model initial and boundary conditions of the traffic flow are defined in Sec. 4.

### Specification-Compliant Scenario Generation

**Fig. 2:**
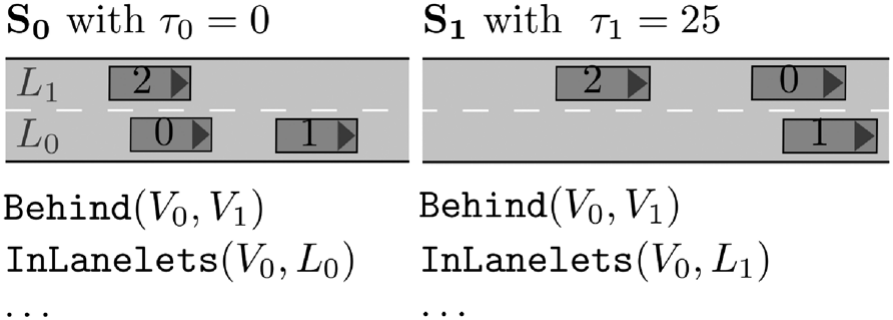
Lane change maneuver of vehicle $V_0$, formally specified by two scenes (similar to [12])

Scenario Factory 2.0 integrates the works in Refs. [12, 13] to generate traffic scenarios from formal specifications. For example, a traffic participant must adhere to the speed limit, is located on a certain lanelet, or is behind another specified traffic participant Ref. [13] (see Fig. 2).

The formal specifications and dynamical vehicle models are converted to linear inequality constraints in a numerical optimization problem. While Ref. [12] solves the optimization problem directly, resulting in trajectories of all traffic participants, Ref. [13] conducts a reachability analysis and allocates conflicting regions of the search space as an intermediate step. Using the latter approach, specifications can be considered that cannot be represented as constraints in a linear optimization problem. Moreover, binary decision variables are eliminated from the optimization problem, resulting in faster execution times.

**Fig. 3:**
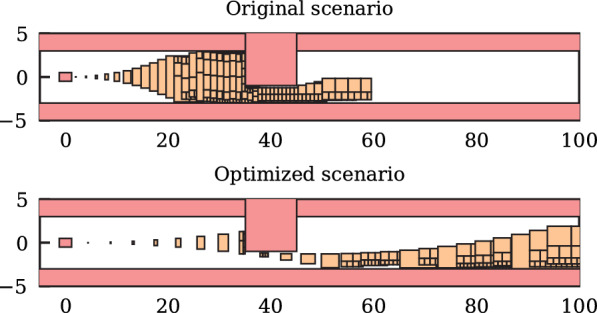
Enhancing scenario criticality by adjusting the initial state to minimize the drivable area [35]

Scenario Factory 2.0 also integrates the work of Ref. [35], where the user specifies a desired drivable area for the vehicle under test. To fulfill this specification, the tool adjusts the initial states of vehicles and static obstacles (see Fig. 3). The drivable area of a traffic participant is derived by projecting its reachable set onto the position domain. A reduced drivable area typically causes an increased criticality of scenarios.

While the mentioned methods are suitable to generate specification-compliant scenarios, provoking failures of the vehicle under test is not their immediate objective. This distinguishes them from falsification-based techniques.

### Falsification-Based Scenario Generation

The falsification approach in Ref. [36] controls the vehicles that surround a vehicle under test. First, feasible control input intervals of the surrounding vehicles are derived based on reachability analysis. Subsequently, a Monte Carlo tree search identifies control inputs within the feasible intervals that provoke a specification violation by the vehicle under test. This procedure is iterated until a failure of the vehicle under test is detected. While the rate of successful falsifications is increased, the computation time is typically longer than in Ref. [35].

### Software Architecture

Scenario Factory 2.0 implements a modular software architecture for easy integration and extensibility of functionalities. It defines several standard modules to process scenarios that can be used interchangeably, e.g., traffic simulation can be executed with either SUMO or OpenTrafficSim. Between the software modules, data is forwarded as batches of scenario containers. The scenario containers always contain a CommonRoad scenario and possibly additional attachments, such as metrics or reference scenarios. For an efficient processing, the modules are automatically executed in parallel for multiple scenarios if possible. The following software modules are available.

- Filter, download, and convert maps from OpenStreetMap to the CommonRoad lanelet network format.
- Simulate scenarios with a tunable deviation from provided ones using the simulation modes (see Sec. 4).
- Synthesis of specification-compliant scenarios (see Sec. 3.3).
- Provoking failures of vehicles under test using falsification techniques (see Sec. 3.4).
- Sample scenarios from real-world recordings using the CommonRoad dataset converter.
- Analyze scenarios with automatic labeling, criticality computation [37], and visualization.
- Apply motion planners to traffic scenarios and evaluate their solutions with cost functions.

Software modules can easily be added and extended: For example, to test another motion planner, only interfaces to read CommonRoad scenarios and planning problems and to return a solution trajectory [25] must be implemented.

To create traffic scenarios with desired properties using Scenario Factory 2.0, users can combine individual software modules. Sec. 5.4 demonstrates a scenario generation that includes lanelet network download, random traffic simulation, and criticality enhancement.

## Simulation Modes

The simulation modes realize a tunable similarity to provided scenarios. Depending on the abstraction level, the simulation modes set the timing, position, velocity, type, shape, destination, and route of created traffic participants (see Table 1). All simulation modes are available for coupling with SUMO and OpenTrafficSim. To streamline the presentation, we focus on the OpenTrafficSim implementation subsequently. It applies the lane change model with relaxation and synchronization (LMRS) [32] as the human driver model. The LMRS extends the IDM+ to handle lane changes. While the default model parameter values for the LMRS provided by OpenTrafficSim are used, users can specify values for each parameter. For the velocity and acceleration bounds, defaults depending on the vehicle type are used. The simulation modes are presented in order of increasing traffic information abstraction.

**Table 1:** Simulation mode configurations for traffic scenario generation with origin destination matrix O/D, lane capacity $\kappa$, average traffic load $\overline{\phi }$, and random value

#	Simulation mode	Timing	Position	Velocity	Vehicle type and shape	Destination	Route
1	Resimulation	Exact	Exact	Exact	Exact	Lanelet	Exact
2	Delay	Delayed	Exact	Exact	Exact	Lanelet	Exact
3	Demand	O/D	Lanelet	Model	Distribution	O/D	Shortest
4	Infrastructure	$\kappa \cdot \overline{\phi }$	Lanelet	Model	Distribution	O/D	Shortest
5	Random		Lanelet	Model	General distribution		Shortest

**Resimulation Mode** For each identified shortcoming of an automated driving system, it is often desired to investigate similar scenarios, e.g., to identify when the shortcoming occurs exactly or whether it has been solved for similar scenarios. This can be realized using the resimulation mode, which sets the timing, position, velocity, route, vehicle type, and shape exactly as in the provided scenario. The Cartesian position and velocity from CommonRoad are transformed into curvilinear coordinates along the centerline of the lane where the vehicle is created in OpenTrafficSim. To route traffic participants, their trajectories in the provided scenario are matched to the lanelet network (details in Appendix B.2). As trajectories in provided scenarios might end inside the road network, their routes are randomly extended until they reach an outgoing lanelet (see demand mode for formal definition) to avoid disappearing of traffic participants. The human driver model parameters, e.g., $s_0$ and *T* in Eq. (2), are sampled from the default distributions provided by OpenTrafficSim [30, 32].

**Delay Mode** As vehicle following models aim to be safe, their provided accelerations lead to abrupt braking in unsafe situations [29]. The delay mode therefore delays creating traffic participants if unsafe traffic situations would arise. To this end, OpenTrafficSim provides a headway checker that evaluates whether a vehicle can be created based on the headway *s* and velocity difference $\Delta v$ between the vehicle to be created and its leading vehicle (see Sec. 2.2 for definitions). A vehicle is created if

4 $$ ( {\underbrace {{\Delta v \le 0}}_{{\begin{array}{*{20}c}    {{\text{slower}}\;{\text{than}}}  \\    {{\text{preceding}}\;{\text{vehicle}}}  \\   \end{array} }} \vee \underbrace {{t_{{{\text{ttc}}}}  \ge 5{\mkern 1mu} {\text {s}}}}_{{\begin{array}{*{20}c}    {{\text{sufficient}}\;{\text{time}}}  \\    {{\text{to}}\;{\text{collision}}}  \\   \end{array} }}} ) \wedge \underbrace {{s \ge s_{{{\text{min}}}} }}_{{\begin{array}{*{20}c}    {{\text{sufficient}}}  \\    {{\text{headway}}}  \\   \end{array} }} $$

using the time to collision (see Sec. 5.2.1 [38])

5 $$\begin{aligned} t_{\text{ttc}} = \frac{s}{\Delta v} \end{aligned}$$

The minimum headway $s_{\text{min}}$ is defined using the velocity of the vehicle to be created $v_{\text{create}}$, a duration $\delta = {1}\,{\text{s}}$, and a distance margin $s_{\text{margin}}={3}\,{\text{m}}$:

6 $$\begin{aligned} s_{\text{min}} = \delta \cdot v_{\text{create}} + s_{\text{margin}} \end{aligned}$$

When a vehicle cannot be created due to the conditions in Eq. (4), it is added to the queue of vehicles to be created. As long as the queue is not empty, it is evaluated whether the first vehicle in the queue can be created in each time step. In the simulation modes presented so far, there is a one-to-one mapping between vehicles in the provided scenario and the simulation. This property is relaxed in the next simulation mode.

**Fig. 4:**
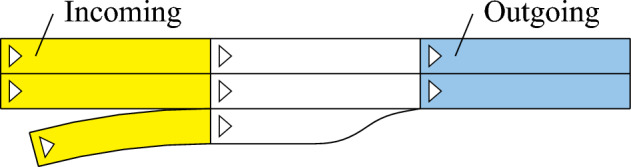
On-ramp lanelet network

**Demand Mode** The demand mode extracts origin-demand (O/D) relations from a provided scenario to simulate new scenarios. To model the O/D relations, incoming and outgoing lanelets are distinguished: Incoming lanelets are those that do not have any predecessor (see yellow lanelets in Fig. 4). Likewise, outgoing lanelets do not have any successor (blue).

Storing O/D relations in O/D matrices and setting the corresponding traffic flow as boundary conditions is a common approach in microscopic traffic simulation (see p.275 [24]). The traffic flow entries $Q_{i,j}$ of the O/D matrix are computed as the number of vehicles $N_{i,j}$ that enter the simulation domain on an incoming lanelet $\square _i$ and leave on an outgoing lanelet $\square _j$ divided by the scenario duration $t_{\text{scenario}}$:

7 $$\begin{aligned} Q_{i,j} = \frac{N_{i,j}}{t_{\text{scenario}}} \end{aligned}$$

Only vehicles are considered that enter the simulation domain after the initial time step and leave it before the end of the traffic scenario. The total traffic flow $Q_i$ of an incoming lanelet $\square _i$ is the sum of traffic flows to all outgoing ones $\square _j$:

8 $$\begin{aligned} Q_i = \Sigma _j Q_{i,j} \end{aligned}$$

In addition to the traffic flows $Q_{i,j}$, the fraction of vehicle types driving from lanelet $\square _i$ to $\square _j$ is stored, as, e.g., trucks might rather take a slow lane on a highway.

The extracted O/D data is fed to so-called generators in OpenTrafficSim that create vehicles based on the information of the O/D matrices. These generators are placed at the beginning of their respective incoming lanelets $\square _i$. The default settings from OpenTrafficSim are used, which set the initial velocity of a vehicle $v_{\text{create}}$ as the minimum of the road speed limit $v_{\text{rsl}}$, the maximum velocity $v_{\text{vsl}}$ of the vehicle, and the velocity of its leading vehicle $v_{\text{lead}}$:

9 $$\begin{aligned} v_{\text{create}} = \min (v_{\text{rsl}}, v_{\text{vsl}}, v_{\text{lead}}) \end{aligned}$$

Given a random value *x* uniformly sampled from [0, 1], OpenTrafficSim sets the desired duration between creating two succeeding vehicles to^2^

10 $$\begin{aligned} \Delta t = -\frac{1}{Q_i} \ln (x) \end{aligned}$$

This modeling guarantees that only positive durations $\Delta t$ are generated and that the expected value corresponds to the traffic flow $Q_i$. The $\ln$-function leads to frequent short intervals and rare large ones, which corresponds to the occurrence of vehicle groups and gaps between them. For creating vehicles during simulation execution, the same delay rules apply as for the delay mode. To determine the route of each vehicle, the demand mode uses the shortest distance route planner provided by OpenTrafficSim. The demand mode applies a warm-up strategy instead of initializing vehicles inside the road network. Therefore, the simulation results are exported as traffic scenarios only after the warm-up duration $t_{\mathrm{warm-up}}$ has passed, as derived subsequently. Let $t_{i,j}$ be the duration of the fastest routes from each incoming $\square _i$ to each outgoing $\square _j$ lanelet (0 if no path exists), assuming that vehicles drive at the road speed limit $v_{\text{rsl}}$. We set five times the longest of these durations as the warm-up duration $t_{\mathrm{warm-up}}$:

11 $$\begin{aligned} t_{\mathrm{warm-up}} = 5 \cdot \max (t_{i,j}) \end{aligned}$$

The factor five accounts for settling phenomena and is assessed in Sec. 5.2.3.

**Infrastructure Mode** The infrastructure mode aims for an increased deviation from the provided scenario, while maintaining realistic traffic flow properties. Specifically, it distributes the total traffic flow of a provided scenario uniformly among all incoming lanelets: Let $Q_i$ be the traffic flow on an incoming lanelet in a provided scenario, and $\kappa _i$ be the traffic flow capacity of that lanelet (see Appendix B.3 for the derivation of the lanelet capacity). We define the average traffic load $\overline{\phi }$ of a scenario as its total flow on all incoming lanelets divided by their total capacity:

12 $$\begin{aligned} \overline{\phi } = \frac{\Sigma _i Q_i}{\Sigma _i \kappa _i} \end{aligned}$$

With the infrastructure mode, the traffic load of the incoming lanelets is equalized, while preserving the fractions for vehicle routing and types, resulting in a modified O/D matrix:

13 $$\begin{aligned} \tilde{Q}_{i,j}^\text{infra} = Q_{i,j} \cdot \frac{\overline{\phi } \cdot \kappa _i}{Q_i} \end{aligned}$$

Except for the modified O/D matrix $\tilde{Q}^\text{infra}$, the simulation is performed as in the demand mode. As the infrastructure mode still requires a provided scenario, we present the random mode next.

**Random Mode** The random mode simulates traffic solely based on a road network. Thus, the scenario generation technique works even if there is no traffic information available in provided scenarios, or if strong deviations are desired. For the random mode, we compute the O/D matrix $\tilde{Q}^\text{rand}$ based on the lane capacity $\kappa _i$ (see Appendix B.3), the load factor $\phi _i$, and the fraction of vehicles $\psi _{i,j}$ that drive from an incoming lanelet $\square _i$ to each reachable outgoing lanelet $\square _j$:

14 $$\begin{aligned} Q_{i,j}^\text{rand} = \kappa _i \cdot \phi _i \cdot \psi _{i,j} \end{aligned}$$

The load factor $\phi _i$ and the fraction of vehicles $\psi _{i,j}$ are sampled from uniform random distributions [0, 1], with $\Sigma _j \psi _{i,j} = 1$. With this random O/D matrix $\tilde{Q}^\text{rand}$, simulations are performed as in the delay mode. Vehicle types are assigned by sampling from random distributions that are calibrated using the MONA dataset [33] (see Table 2).

**Table 2:** Vehicle type distributions in the MONA dataset

Vehicle type $\tau$	$\mu$	$\sigma$
Car	0.865	0.033
Truck	0.112	0.030
Bus	0.005	0.003
Motorcycles	0.009	0.006

## Evaluation

For evaluating Scenario Factory 2.0, the simulation modes (Sec. 5.2) and the computational performance of the OpenTrafficSim coupling (Sec. 5.3) are assessed. In addition, a use case (Sec. 5.4) is presented that demonstrates our contributions. First, the datasets used in the evaluation are introduced.

### Scenarios

**Fig. 5:**
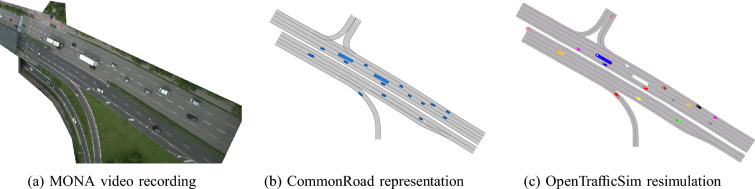
Representations of the MONA West traffic scenario

We evaluate the simulation modes with recorded scenarios provided from the inD [39], highD [5], and MONA [33] datasets. With the CommonRoad dataset converter,^3^ all datasets are converted to the CommonRoad format and various traffic situations are selected, e.g., urban intersections, highway driving, and main roads with varying traffic loads. All scenarios are cut to a duration of 60 s. Shorthand notations and the respective CommonRoad scenario IDs are shown in Table 3. Different stages of the MONA West scenario are visualized in Fig. 5.

**Table 3:** Shorthand notation of scenarios used in the evaluation

Shorthand	CommonRoad scenario ID
inD	DEU_AachenBendplatz-1_90_T-300
highD	DEU_LocationCLower6-1_60_T-300
MONA East	DEU_MONAEast-2_1_T-300
MONA Merge	DEU_MONAMerge-2_1_T-300
MONA West	DEU_MONAWest-2_1_T-300

### Simulation Modes

Depending on the simulation mode, different metrics are appropriate to assess their resulting properties. For the resimulation and delay modes, trajectories of individual vehicles are compared to the provided real-world scenarios (see Sec. 5.2.1). In addition, the effect of creation delay is investigated (see Sec. 5.2.2). For simulation modes that use a warm-up phase, it is evaluated whether its duration is sufficient for settling processes to be completed (see Sec. 5.2.3). Moreover, all simulation modes are compared applying macroscopic traffic metrics (see Sec. 5.2.4).

#### Trajectory Comparison

A common traffic similarity metric on a microscopic scale is the root mean squared error (RMSE) [40]. Cumulative variables, such as the position compared to the acceleration, are preferable due to their high information content [41]. Therefore, we compute the RMSE for each vehicle based on the Euclidean distance $\Vert \cdot \Vert$ between its simulated position $p^\text{sim}$ and the provided position $p^\text{pro}$, with *T* being the number of evaluated time steps:

15 $$\begin{aligned} {{RMSE}} = \sqrt{\frac{1}{T} \cdot \sum _{k=1}^T \Vert p^{\text{sim}}(k) - p^{\text{pro}}(k) \Vert ^2} \end{aligned}$$

For its evaluation, the RMSE data is further aggregated: The index $\square _{\mu }$ denotes the mean RMSE of all vehicles in a scenario and $\square _{\sigma }$ the respective standard deviation. Together with the other metrics, the results are listed in Table 4. High standard deviations ${{RMSE}}_\sigma$ compared to mean values ${{RMSE}}_\mu$ indicate a wide spread of trajectory similarity for the individual vehicles. For many scenarios, low ${{RMSE}}_\mu$ values ($\sim {10}\,{\text{m}}$) occur, indicating a high similarity between simulated and provided trajectories. As intended, the ${{RMSE}}_\mu$ of the delay mode is typically increased, indicating a reduced similarity with the provided scenarios.

**Table 4:** Values of various metrics for each simulation mode using OpenTrafficSim simulations; if a field is empty, its metric is not applicable; results for SUMO simulations are in Table A1

Sim. mode	${\text{RMSE}}$		*f*	$\rho$		*v*	
	$\mu$	$\sigma$		$\mu$	$\sigma$	$\mu$	$\sigma$
	(m)	(m)	($\hbox {s}^{-1}$)	($\hbox {km}^{-1}$)	($\hbox {km}^{-1}$)	(${\text{m}}\cdot{\text{s}}^{-1}$)	(${\text{m}}\cdot{\text{s}}^{-1}$)
inD							
*Recorded*			0.29	6.49	4.87	6.69	4.66
Resim	7.04	6.94	0.29	5.32	3.53	9.13	2.68
Delay	5.85	5.55	0.29	5.41	3.62	8.60	3.12
Demand			0.28	4.06	1.81	10.93	1.73
Infrastr			0.27	9.86	2.12	4.44	1.21
Random			0.58	22.25	6.01	3.35	0.94
highD							
*Recorded*			0.42	4.07	1.44	31.95	3.22
Resim	12.39	10.83	0.42	4.05	1.32	32.29	2.47
Delay	23.27	31.45	0.42	3.64	1.39	32.92	3.02
Demand			0.40	3.80	0.81	34.83	1.51
Infrastr			0.51	4.94	0.92	33.77	1.23
Random			0.64	7.74	1.54	27.83	0.97
MONA East							
*Recorded*			1.87	13.82	1.63	11.06	1.61
Resim	7.45	8.59	1.85	11.47	1.54	13.29	2.92
Delay	11.85	15.90	1.85	11.08	1.63	15.30	0.55
Demand			1.68	14.09	1.06	11.40	0.54
Infrastr			1.69	17.53	1.60	6.89	0.54
Random			1.42	17.10	1.19	6.72	0.73
MONA Merge							
*Recorded*			0.77	9.53	4.90	18.58	1.97
Resim	7.26	7.27	0.77	12.79	3.17	14.42	3.61
Delay	14.39	18.49	0.77	11.34	3.43	16.83	2.18
Demand			0.72	11.68	2.15	16.42	0.62
Infrastr			0.67	11.32	2.80	15.70	0.73
Random			0.48	7.70	2.45	16.49	0.83
MONA West							
*Recorded*			1.47	10.56	3.66	15.12	0.85
Resim	7.22	5.49	1.48	8.76	3.45	13.76	1.29
Delay	16.11	23.65	1.46	8.60	3.04	13.96	1.07
Demand			1.65	12.97	1.77	14.79	2.29
Infrastr			1.52	14.24	2.72	12.74	0.90
Random			0.76	13.69	5.14	4.37	1.28

Other common metrics in the field of motion prediction stem from the Waymo open dataset challenge [9]. To enhance comparability to other works, we provide the respective metrics (see Table 5). While the Waymo motion prediction challenge selects the best one of six trajectory predictions to compute the metrics, our approach only provides one prediction, which explains the seemingly deteriorated performance.

**Table 5:** Waymo metrics at $t={5}\,{\text{s}}$: average displacement error (ADE5); final displacement error (FDE5); miss rate (MR5)

Scenario	ADE5 (m)	FDE5 (m)	MR5 (%)
inD	3.71	8.45	14.9
highD	4.26	7.33	27.4
MONA East	2.61	5.98	15.4
MONA Merge	5.39	12.22	7.3
MONA West	3.69	7.70	20.7

#### Delay

**Fig. 6:**
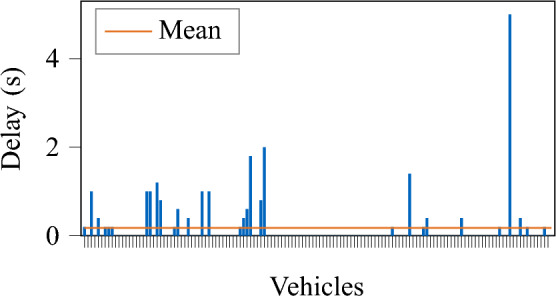
Delay of created vehicles in the MONA East scenario; vehicles are sorted according to their time of creation in the provided scenario

For many vehicles, there is no or only minor delay, and there are some outliers (see Fig. 6). These delays are correlated with the occurrence of traffic jams that hinder the creation of vehicles in the simulation.

#### Warm-up Duration

**Fig. 7:**
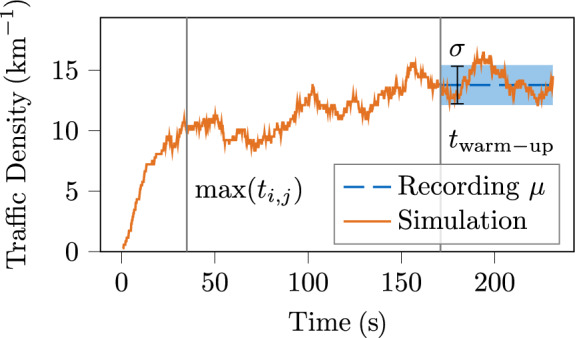
Traffic density $\rho (t)$ in demand mode simulation of MONA East scenario

**Fig. 8:**
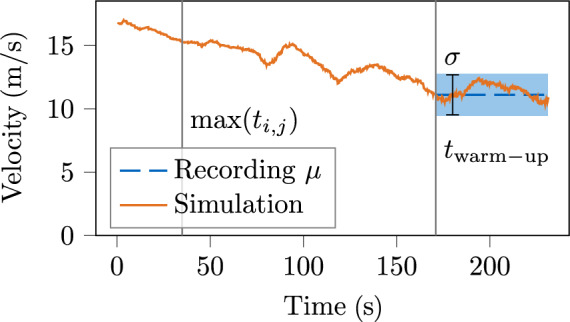
Mean velocity $\overline{v}(t)$ of vehicles in demand mode simulation of MONA East scenario

We first demonstrate settling processes in an example using two metrics. Subsequently, those findings are generalized for other simulations. Let *n*(*t*) be the number of vehicles in a simulation at a time *t* and *L* the total length of its lanelet network. The traffic density $\rho (t)$ is given by (see Eq. (11) [29])

16 $$\begin{aligned} \rho (t) = \frac{n(t)}{L} \end{aligned}$$

and the mean velocity $\overline{v}(t)$ of vehicles in the simulation domain by

17 $$\begin{aligned} \overline{v}(t) = \frac{1}{n(t)} \sum _{i=1}^{n(t)} v_i(t) \end{aligned}$$

with the velocity of the i$^{th}$ vehicle $v_i(t)$. The index $\square _{\mu }$ denotes the time average of the traffic density $\rho (t)$ and the mean velocity $\overline{v}(t)$, while $\square _{\sigma }$ denotes the respective standard deviation. For the MONA East scenario, the estimated maximum duration for vehicles to travel the whole road network (see Sec. 4, Delay Mode) is $\max (t_{i,j}) = {34.2}\,{\text{s}}$. In fact, the simulated traffic density $\rho (t)$ in the demand mode stabilizes after approximately this duration (see Fig. 7), as the frequency of vehicles entering and leaving the simulation domain converges to an equilibrium. Due to the transient and interfering nature of traffic, the mean velocity of vehicles $\overline{v}(t)$ does not converge until then (see Fig. 8). The duration is extended in Eq. (11), and simulation results are exported only after $t_{\mathrm{warm-up}} = {170.4}\,{\text{s}}$ to ensure that settling processes are completed. By that time, the simulated traffic density $\rho (t)$ and mean velocity $\overline{v}(t)$ consistently fluctuate around the respective mean values of the provided real-world scenario.

The convergence of the aggregated metrics towards the mean values thus provides an alternative indicator when a settling process is completed. The simulated traffic density $\rho _\mu$ and the mean velocity $\overline{v}_\mu$ must lie within an acceptable range. In fact, $\rho _\mu$ and $\overline{v}_\mu$ in demand mode typically lie within the standard deviation from the mean values of the provided real-world scenarios.

#### Comparison of Simulation Modes

Next, we define the mean creation frequency *f* and compare the simulation modes in terms of their macroscopic traffic metrics. The mean creation frequency *f* is the ratio of the number of vehicles $N_\text{Scenario}$ that are created during a scenario and its duration $t_\text{Scenario}$:

18 $$\begin{aligned} f = \frac{N_\text{Scenario}}{t_\text{Scenario}} \end{aligned}$$

While the mean creation frequency *f* of resimulations is identical to provided scenarios, it can be slightly smaller for the delay mode (see Table 4). The mean creation frequency *f* increasingly deviates for the demand, infrastructure, and random modes.

A fundamental correlation of human driver models is reflected in the simulated traffic scenarios: High traffic densities $\rho _\mu$, and thus short longitudinal distances $\overline{s}$ between succeeding vehicles, lead to reduced traffic velocities $v_\mu$ (see Eqs.(2) and (3) [30]). The deviations of both quantities typically increase with higher abstraction levels. The standard deviations are often reduced for the simulations, suggesting less heterogeneity of traffic.

The similarity of resimulated scenarios, indicated by the metrics, confirms them to be realistic, i.e., close to the provided real-world scenarios. The metrics also indicate an increased deviation from the recordings with reduced information extraction by the simulation modes. Thus, the desired similarity of generated scenarios is tunable by selecting appropriate simulation modes.

Complementary to the OpenTrafficSim results in Table 4, the SUMO results are provided in Table A1. While individual values differ due to different human driver models, random elements, and deviating modeling details, the overall characteristics are identical. However, the resimulation and delay modes show a closer similarity for the OpenTrafficSim than for the SUMO simulations - probably due to the more refined human driver models.

### Computational Performance

The various simulation modes perform different pre-processing steps and execute the simulation for deviating durations, influencing the computation times (see Tab 6). All timings are conducted with an Intel i7-12700 H processor. The matching of trajectories to the lanelet network conducted for resimulation and delay modes extends their pre-processing time. Simulation modes using the warm-up strategy have an increased simulation time. The export of simulation results to CommonRoad is similar for all simulation modes. Overall, the scenario generation takes about ${2}\,{\text{s}}$, while the scenarios have a duration of ${60}\,{\text{s}}$. Thus, it is about 30 times faster than real time. Moreover, with the modular software architecture, simulations are automatically parallelized for several scenarios. These fast execution speeds make the scenario generation using simulation modes well suited for large-scale test generation.

**Table 6:** Execution times averaged over all simulations conducted for the evaluation (s)

Sim. mode	Conversion	Simulation	Export	$\Sigma$
Resim.	0.51	1.01	0.17	1.70
Delay	0.52	0.85	0.15	1.52
Demand	0.11	1.63	0.18	1.92
Infrastr.	0.18	1.69	0.19	2.06
Random	0.10	1.98	0.34	2.43

### Use Case

**Fig. 9:**
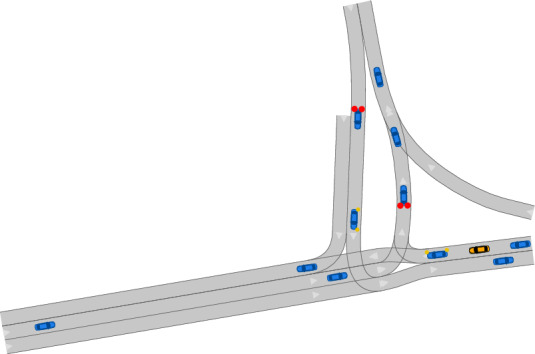
Use case of traffic scenario generation at an extra-urban intersection; the tested vehicle (orange) drives straight while its preceding vehicle turns right

The evaluation of the OpenTrafficSim coupling and its simulation modes is complemented by showcasing the applicability of Scenario Factory 2.0 on a use case. First, we specify a location near the TUM campus in Garching, Germany. Based on this input, eight interesting lanelet networks including intersections and highway configurations in a radius of 400 m are downloaded as OpenStreetMap files and converted to CommonRoad lanelet networks. Subsequently, random traffic is simulated with SUMO on these lanelet networks. Multiple traffic scenarios with a predefined duration of 15 s are derived by analyzing the resulting trajectories for interesting maneuvers. Corresponding labels are assigned such that the scenarios can be easily filtered and used for motion planner testing. Next, the time to collision criticality metric Eq. (5) is computed for the tested vehicles. We select a rather uncritical scenario with an initial time to collision $t_\text{TTC} = {2.42}\,{\text{s}}$ (see Table II [42]). In this scenario, the preceding vehicle decelerates to turn right, whereas the testing vehicle drives straight (see Fig. 9). The criticality enhancement tool [35] adjusts the initial velocity of the tested vehicle, reducing the time to collision $t_\text{TTC} = {0.17}\,\hbox {s}$. Using the resimulation mode, a further test case with a time to collision $t_\text{TTC} = {0.87}\,\hbox {s}$ is obtained, providing a compromise between realistic and critical properties. This example demonstrates the versatility of Scenario Factory 2.0 for scenario-based testing of autonomous vehicles.

## Conclusion and Outlook

By seamlessly integrating several techniques for scenario generation, Scenario Factory 2.0 offers a valuable contribution to the scenario-based testing of automated vehicles. The novel simulation modes extract traffic information from provided scenarios and support coupling with SUMO and OpenTrafficSim. They are well suited to simulate traffic scenarios with desired similarity. Simulation modes that create similar scenarios to ones that are known to cause failures are useful for systematically analyzing automated driving systems. With highly deviating traffic scenarios, users can quickly search for unknown shortcomings. Further synergies are leveraged when combining the simulation-based approach with other techniques, such as synthesis from formal specifications or criticality enhancement methods. The design of Scenario Factory 2.0 defines software modules for scenario generation and utility functionalities, and achieves a previously unseen flexibility for the combination of scenario generation techniques. This directly addresses the problem that existing approaches typically solve specific applications and are limited in their compatibility with each other. We demonstrate the functionality and flexibility of Scenario Factory 2.0 in a use case. Being published as an open-source software, Scenario Factory 2.0 contributes to the development and validation of automated driving systems using scenario-based testing. We will continuously improve Scenario Factory 2.0, supporting additional features and responding to feedback from the autonomous driving research community.

## Appendix A: Additional Results

The results for the SUMO simulations complementary to Table 4 are provided in Table A1.

**Table A1:** Values of various metrics for each simulation mode using SUMO simulations; if a field is empty, its metric is not applicable

Sim. mode	${\text{RMSE}}$		*f*	$\rho$		*v*	
	$\mu$ (m)	$\sigma$ (m)		$\mu$ ($\hbox {km}^{-1}$)	$\sigma$ ($\hbox {km}^{-1}$)	$\mu$ (${\text{m}}\cdot{\text{s}}^{-1}$)	$\sigma$ (${\text{m}}\cdot{\text{s}}^{-1}$)
			($$\hbox {s}^{-1}$$)				
inD							
*Recorded*			0.29	6.49	4.87	6.69	4.66
Resim	14.60	7.25	0.27	3.86	3.18	7.93	2.13
Delay	18.02	14.48	0.27	3.67	3.09	8.00	1.73
dem			0.19	2.92	1.83	11.49	3.25
Infrastr			0.23	3.49	2.35	11.25	3.51
Random			0.67	15.67	6.82	6.08	2.25
highD							
*Recorded*			0.42	4.07	1.44	31.95	3.22
Resim	18.16	9.46	0.42	3.34	1.37	36.08	2.43
Delay	19.13	11.42	0.42	3.34	1.37	36.09	2.44
dem			0.30	2.48	0.91	33.63	2.07
Infrastr			0.29	2.34	0.78	33.98	1.57
Random			0.67	5.61	1.94	33.42	2.24
MONA East							
*Recorded*			1.87	13.82	1.63	11.06	1.61
Resim	28.01	17.09	1.85	10.54	1.53	14.89	3.00
Delay	27.07	20.52	2.02	9.10	2.11	17.98	1.96
dem			1.80	11.88	3.17	12.93	1.01
Infrastr			1.86	9.67	2.44	14.18	0.61
Random			1.27	8.43	2.01	12.35	0.83
MONA Merge							
*Recorded*			0.77	9.53	4.90	18.58	1.97
Resim	10.96	4.40	0.77	7.42	3.50	17.41	2.77
Delay	19.51	22.57	0.81	6.97	3.56	18.68	1.04
dem			0.71	11.50	5.61	14.39	1.81
Infrastr			0.71	11.48	4.83	14.59	0.41
Random			0.46	7.50	1.84	15.45	0.93
MONA West							
*Recorded*			1.47	10.24	3.55	15.12	0.85
Resim	13.73	14.20	1.50	9.72	2.50	14.15	1.43
Delay	32.02	32.72	1.50	10.14	3.07	13.79	1.40
dem			1.25	11.88	3.52	11.27	4.94
Infrastr			1.14	10.59	3.00	10.58	3.98
Random			1.57	13.58	3.00	11.93	1.01

## Appendix B: Implementation Details

### B.1 Traffic Signs and Lights

While CommonRoad models traffic signs and lights as physical objects, OpenTrafficSim captures their effects instead. To accurately capture the effect of persistent traffic signs, such as speed limit signs, their validity for succeeding lanelets is pre-processed. Some traffic signs, e.g., no overtaking, are currently not implemented in OpenTrafficSim, limiting the conversion accuracy in special cases. In CommonRoad, traffic lights including their validity direction are assigned to the last lanelets before an intersection. Using the intersection information contained in CommonRoad scenarios, CommonRoad traffic lights are converted into the OpenTrafficSim representation by assigning the correct traffic light cycles to the lanes succeeding the traffic lights.

### B.2 Matching Trajectories to Lanelet Networks in CommonRoad

OpenTrafficSim currently does not support overtaking parked vehicles by driving on oncoming lanelets. Therefore, we ignore parked vehicles and those that cannot be matched successfully to the lanelet network during the conversion process. For matching the trajectory of a vehicle to a lanelet network, candidate lanelet sequences are generated that connect the lanelets that the vehicle intersects in its initial and final state. Of those candidates, the lanelet sequence with the highest number of trajectory states that intersect with the lanelet sequence is selected.

### B.3 Derivation of Traffic Flow Capacity of a Lanelet

To compute the capacity $\kappa _i$ of a lanelet $\square _i$, we assume equilibrium traffic conditions ($\dot{v}=0$, $\Delta v=0$) [29]. As the vehicle velocity *v*, gap size *s*, and vehicle length *l* are strictly positive values, the traffic flow *Q* in Eq. (3) increases with high velocities *v* and small gap sizes *s*. However, Eq. (1) limits the vehicle velocity $v \le v_0$ and gap size $s \ge s^*$ in equilibrium traffic conditions, such that the desired minimum gap size (2) becomes

B1 $$\begin{aligned} s^* = s_0 + v \cdot T \end{aligned}$$

Inserting $s = s^*$ into Eq. (3) yields the traffic flow:

B2 $$\begin{aligned} Q = \frac{v}{l + s_0 + v \cdot T} \end{aligned}$$

With *l*, $s_0$, and *T* being fixed parameters, its derivative with respect to the velocity is

B3 $$\begin{aligned} \frac{{\text{d}}Q}{{\text{d}}v} = \frac{l + s_0}{(l + s_0 + v \cdot T)^2} > 0 \end{aligned}$$

Thus, the maximum traffic flow in equilibrium conditions is reached for the maximum admissible vehicle velocity $v = v_0$:

B4 $$\begin{aligned} \kappa _i = \frac{v_0}{l + s_0 + v_0 \cdot T} \end{aligned}$$

We use the default parameters of the IDM+ model for $s_0 = {2}\,\hbox {m}$ and $T = {1.45}\,\hbox {s}$ (see Fig. 1a [30]), while the speed limit of the lanelet is set as the desired velocity $v_0$. Moreover, the length *l* of a lanelet is averaged over all vehicles that are created on that lanelet.

## Footnotes

1 https://commonroad.in.tum.de/scenarios

2 https://github.com/averbraeck/opentrafficsim/blob/3d6bc7d8c820287a0df0a5c4f8aeee1f851a1509/ots-road/src/main/java/org/opentrafficsim/road/gtu/generator/headway/HeadwayGenerator.java#44

3 https://commonroad.in.tum.de/tools/dataset-converters
